# A DEDICATED APPROACH TO PALLIATIVE DEMENTIA CARE

**DOI:** 10.1093/geroni/igac059.2032

**Published:** 2022-12-20

**Authors:** Judith Meijers, Sascha Bolt, Sandra Zwakhalen, Chandni Khemai, Jos Schols, Daisy Janssen, Jenny van der Steen, Jesper Biesmans

**Affiliations:** Maastricht University, Maastricht, Limburg, Netherlands; Tilburg University, Tilburg, Noord-Brabant, Netherlands; Maastricht University, Maastricht, Limburg, Netherlands; Maastricht University, Maastricht, Limburg, Netherlands; Maastricht University, Maastricht, Limburg, Netherlands; Maastricht University, Maastricht, Limburg, Netherlands; Leiden University Medical Center, Leiden, Zuid-Holland, Netherlands; Zuyderland, Zuyderland, Limburg, Netherlands

## Abstract

Nursing staff play a central role in the palliative care for people with dementia. Development of their palliative care competences may support timely recognition and addressing of individual needs of persons with dementia and their family caregivers in long term care. In the DEDICATED (Desired Dementia Care Towards End of Life) project, we aim to develop materials to support nursing staff in providing palliative dementia care. The first step of the project concerned a needs assessment, mapping the perspectives of nursing staff, family caregivers and people with dementia (scoping review, surveys and semi-structured interviews). Using these studies’ results as a starting point, an intervention (the DEDICATED approach) was built, using an iterative co-creation approach involving nursing staff and educators (N=12). These ‘ambassadors’ were also trained to disseminate the approach within their care teams and nursing curricula. First reactions from the pilot study regarding the DEDICATED-materials are positive and nurses and nurse educators are eager to use the materials (www.dedicatedwerkwijze.nl). Currently the DEDICATED-approach is being evaluated using a mixed methods pretest-posttest controlled design comparing three groups: a design group (12 wards), a test group (28 wards, 28 new-trained ambassadors) and a control group (12 wards). The main outcomes of study are e.g. self-efficacy, empowerment and engagement in providing palliative care for people with dementia. A secondary outcome involves longitudinal trends of the quality of dying of persons with dementia within the group.

